# *Mentha longifolia* Essential Oil and Pulegone in Edible Coatings of Alginate and Chitosan: Effects on Pathogenic Bacteria in Lactic Cheese

**DOI:** 10.3390/molecules28114554

**Published:** 2023-06-05

**Authors:** Fatemeh Shahdadi, Maliheh Faryabi, Haroon Khan, Ali Salehi Sardoei, Bahman Fazeli-Nasab, Bey Hing Goh, Khang Wen Goh, Ching Siang Tan

**Affiliations:** 1 Food Science and Technology Department, Faculty of Agriculture, University of Jiroft, Jiroft 7867155311, Iran; 2 Faculty of Basic Sciences, Jiroft Branch, Islamic Azad University, Jirof 5716963896, Iran; 3 Department of Pharmacy, Abdul Wali Khan University Mardan, Mardan 23200, Pakistan; haroonkhan@awkum.edu.pk; 4 Faculty of Plant Production, Gorgan University of Agricultural Sciences and Natural Resources, Gorgan 4918943464, Iran; 5 Department of Agronomy and Plant Breeding, Agriculture Institute, Research Institute of Zabol, Zabol 9861335856, Iran; 6 Department of Biotechnology and Plant Breeding, Faculty of Agriculture, Ferdowsi University of Mashhad, Mashhad 9177948978, Iran; 7 School of Pharmacy, Monash University Malaysia, Bandar Sunway 47500, Malaysia; 8 College of Pharmaceutical Sciences, Zhejiang University, Hangzhou 310058, China; 9 Faculty of Data Science and Information Technology, INTI International University, Nilai 71800, Malaysia; khangwen.goh@newinti.edu.my; 10 School of Pharmacy, KPJ Healthcare University College, Nilai 71800, Malaysia

**Keywords:** antibacterial, chitosan, alginate, pulegone, natural product, medicinal and food plants, phytochemicals, bioactive compounds

## Abstract

*Mentha longifolia* is a valuable medicinal and aromatic plant that belongs to *Lamiaceae* family. This study looked at the antibacterial effects of *M. longifolia* essential oil and pulegone in edible coatings made of chitosan and alginate on the growth of *Staphylococcus aureus*, *Listeria monocytogenes*, and *Escherichia coli* in cheese. For this purpose, first fresh mint plant was collected from the cold region of Jiroft in Kerman province. Plant samples were dried in the shade at ambient temperature, and essential oil was prepared using Clevenger. The essential oil was analyzed by gas chromatography using mass spectrometric (GC/MS) detection. The major composition of *M. longifolia* oil was pulegone (26.07%), piperitone oxide (19.72%), and piperitone (11.88%). The results showed that adding *M. longifolia* essential oils and pulegone to edible coatings significantly reduced the growth of bacteria during storage. The bacterial population decreased by increasing the concentration of chitosan, *M. longifolia*, and pulegone in edible coatings. When the effects of pulegone and *M. longifolia* essential oils on bacteria were compared, it was found that pulegone had a stronger effect on bacterial population reduction. Coating treatments showed more antibacterial activity on *E. coli* than other bacteria. In general, the results of this research showed that alginate and chitosan coatings along with *M. longifolia* essential oil and its active ingredient pulegone had antibacterial effects against *S. aureus*, *L. monocytogenes*, and *E. coli* in cheese.

## 1. Introduction

Contaminated milk and dairy products are one of the important sources of infections and food intoxication in humans. One of the causes of contamination of dairy products is their suitable conditions for the growth of many microorganisms. In cheese, depending on the preparation stages and ripening condition, there is a possibility of contamination with various spoilage and disease-causing bacteria, including *E. coli*, *S. aureus*, *L. monocytogenes*, etc. *E. coli* is part of the natural intestinal flora of all warm-blooded animals. The presence of this bacterium in water and food is accepted as an indicator of fecal contamination and the possible presence of dominant pathogens [1]. *S. aureus* is the third cause of foodborne diseases in the world. This bacterium exists in the mammary ducts of cows suffering from mastitis and enters the milk during milking, and the use of this milk in the production of traditional cheese causes its contamination. Food intoxication with *S. aureus* occurs as a result of consuming enterotoxin-contaminated food, and it can remain in cheese for a long time [2]. *L. monocytogenes* is a facultative intracellular pathogenic organism to humans and animals. This is the cause of listeriosis infection in humans and animals. Symptoms of the disease in humans include abortion, encephalitis, meningitis, and septicemia (especially in people with a weak immune system) [3]. Traditional milk products such as cheese, ice cream, etc., which are prepared from unpasteurized milk, can carry this bacteria [4].

Today, people turn to herbal medicines for various reasons, including the high cost of chemical drugs and their side effects. Numerous studies have been conducted to find natural compounds with biological activities derived from plant, animal, and microbial resources because, on the one hand, common chemical drugs used to treat infectious diseases are becoming increasingly ineffective against microorganisms, and, on the other hand, consumers are becoming more aware of the negative effects of chemical and synthetic food preservatives [5]. Medicinal plants are important sources of antimicrobial agents [6,7]. To date, more than 28,187 medicinal species are used by humans. There are over 13,400 plants with defined antimicrobial activity, and over 30,000 antimicrobial compounds have been isolated from plants [8]. Essential oils and extracts have natural antimicrobial activity against a large number of spoilage and pathogenic bacteria; most of these compounds have phenolic active groups in their structure [9]. These compounds can act as an active ingredient, a flavoring, and as a preservative (antibacterial) in food [10]. Some plant families, such as *Lamiaceae*, have not only antimicrobial but also antioxidant properties compared to other families [11].

*Lamiaceae* is a family of plants that includes *Mentha longifolia*. In temperate areas of central and southern Europe, southwest Asia, and Australia, it primarily grows wild in wet environments beside rivers and other waterways [12,13]. Research shows that the essential oils of the *Lamiaceae* are cyclohexanes and aromatic, and pulegone as the main constituent of *M. longifolia* oils has a specific aroma ranging from intense to spicy and vinegar. The main constituents of *M. longifolia* essential oil are different in various studies. In several studies, the major constituents were pulegone, cineole, linalool, menthol, carvone, piperthone, thymol, beta-caryophyllene, etc. These compounds have antimicrobial properties [14,15].

In a study, it was found that *M. longifolia* essential oil had a great inhibitory effect on *L. monocytogenes*, *Klebsiella pneumoniae*, fungi and yeasts, and *E. coli* was the least sensitive [16]. It has also been reported that the essential oil of *M. longifolia* from different regions of Lorestan province in Iran exhibited high inhibitory effect against *Pseudomonas aeruginosa*, *S. aureus*, and *E. coli* [17].

Edible coatings as a consumable layer have adhesive, antioxidant, and antibacterial properties. By covering the surface of the product, these coatings prevent the reduction of moisture and the penetration of oxygen, and improve the appearance of the product [18]. Many organic substances with antioxidant or antibacterial properties, such as plant essential oils and extracts, are added to edible coatings [19]. The use of edible coatings to carry plant essential oils can reduce the negative effects of essential oils such as severe aroma and organoleptic changes [20]. Various materials are used as edible coatings. Alginate is obtained from the cell wall of brown seaweed *Marocystis pyrifera*. Alginates are considered emulsifiers, stabilizers, and thickeners [21]. When a thin layer of gel or alginate solution dries, a film or coating forms that can maintain water-holding capacity, protect against microbial spoilage, and resist oxidation [22].

Because chitosan is effective at preventing the growth of Gram-positive and Gram-negative bacteria, yeasts, and molds, it is used as an edible film and coating. Chitosan has natural antioxidant and antimicrobial properties that are affected by its concentration and molecular weight [23]. Due to the increasing tendency to use natural preservatives, the objective of this study was to determine whether adding *M. longifolia* essential oil and pulegone to edible coatings made of alginate and chitosan would affect the growth of *S. aureus*, *L. monocytogenes*, and *E. coli* in cheese.

## 2. Results

### 2.1. Chemical Compounds of M. longifolia Essential Oil

The *M. longifolia* essential oil compounds were identified using GC/MS. Table 1 shows the identified chemical compounds and the percentage of each component in the essential oil. According to the obtained results, 40 compounds were identified in *M. longifolia* essential oil, which comprised 97.8% of the total essential oil. The main compounds of the essential oil are pulegone (26.07%), piperitone oxide (19.72%), piperitone (11.88%), 1,8-cineole (8.21%), cis-piperitone oxide (6.35%), and borneol (5.96%). As Table 1 shows, *M. longifolia* essential oil is mainly composed of monoterpene compounds (pulegone, piperitone oxide, and piperitone).

### 2.2. Descriptive Statistics Related to the Chitosan, Pulegone and Essential Oil on E. coli and S. aureus (cfu/g) in Cheese during Storage

Descriptive statistics related to chitosan, pulegone, and essential oil in *E. coli*, *S. aureus*, and *L. monocytogenes* are presented in Table 2, Table 3, Table 4, Table 5, Table 6 and Table 7. Based on the values of standard deviation, minimum, maximum, and mean, The 30th day and The first day had the least and the most variation, respectively. The results showed that the treatments under different concentrations have different characteristics.

### 2.3. The Effect of Various Concentrations of Chitosan and M. longifolia Essential Oil on E. coli (cfu/g) in Cheese during Storage

According to Table 8 findings, the *E. coli* population in cheese reduced when storage period, chitosan concentration, and essential oil levels were increased. At the conclusion of the storage time, the lowest number of bacteria related to the treatment contained 10% chitosan and 150 ppm peppermint essential oil. On all days of storage, the control showed the greatest level of bacterial population.

**Figure 1:**
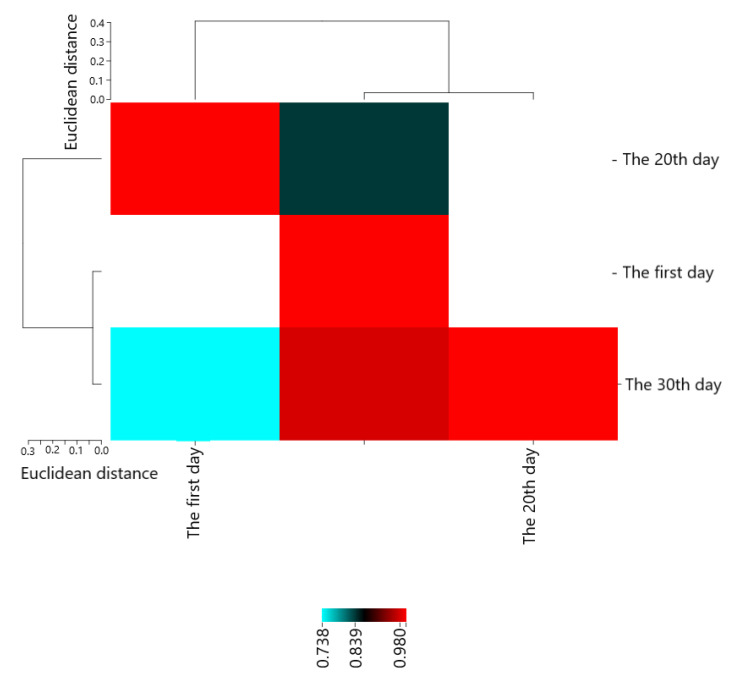
Heat map of mutual relations of chitosan and essential oil on *E. coli* (cfu/g) in cheese during storage in correlation coefficient.

Pearson’s correlation coefficients are presented in Figure 1. The results showed that The first day with The 20th day (0.83927 *), The 30th day (0.97960 **), and The 20th day with The 30th day (0.73614 *) had the most positive and significant correlation.

### 2.4. The Effect of Various Concentrations of Chitosan and Pulegone on E. coli (cfu/g) in Cheese during Storage

The results of Table 9 show that with increasing pulegone concentration, the number of *E. coli* bacteria in cheese decreased. As the storage period increased, the population of *E. coli* in cheese samples coated with pulegone and chitosan decreased. On the first and 20th days of storage, increasing the amount of chitosan from 5 to 10% in the coatings did not have a significant effect on the bacterial population. At the conclusion of the storage time, the control and treatment coated with 10% chitosan and 50 ppm pulegone had the greatest and lowest populations of bacteria, respectively.

**Figure 2:**
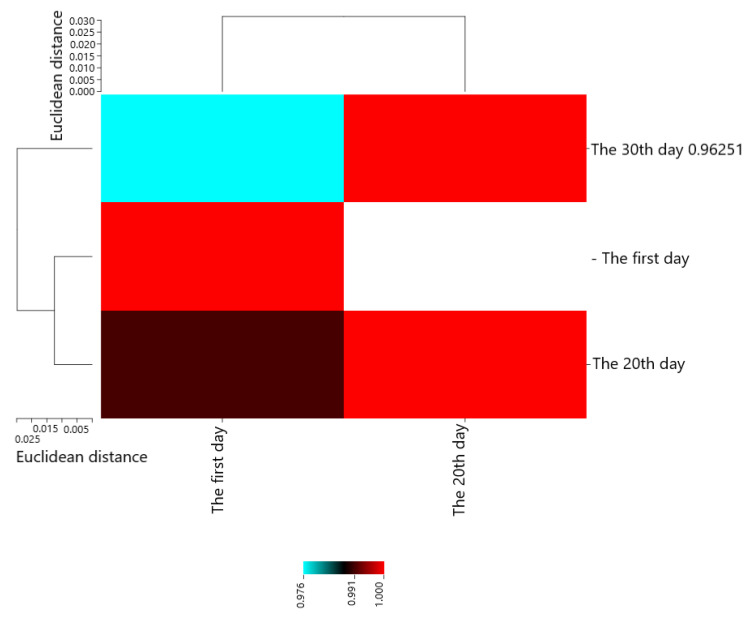
Heat map of mutual relations of chitosan and pulegone on *E. coli* (cfu/g) in cheese during storage in correlation coefficient.

Pearson’s correlation coefficients are presented in Figure 2. The results showed that The first day with The 20th day (0.99118 **), The 30th day (0.96251 **), and The 20th day with The 30th day (0.97579 **) had the most positive and significant correlation.

### 2.5. The Effect of Various Concentrations of Chitosan and M. longifolia Essential Oil on S. aureus (cfu/g) in Cheese during Storage

According to Table 10, it can be concluded that the population of *S. aureus* in cheese reduced with longer storage times in all treatments. At the conclusion of the storage time, the lowest number of bacteria related to the treatment contained 10% chitosan and 150 ppm essential oil. The treatment coated with 5% chitosan without essential oil had the most bacteria population, but there was no significant difference between this treatment, control and the treatment coated with 10% chitosan without essential oil (*p* > 0.05). By increasing the amount of essential oil and chitosan in the coatings, the population of *S. aureus* in the cheese samples decreased.

**Figure 3:**
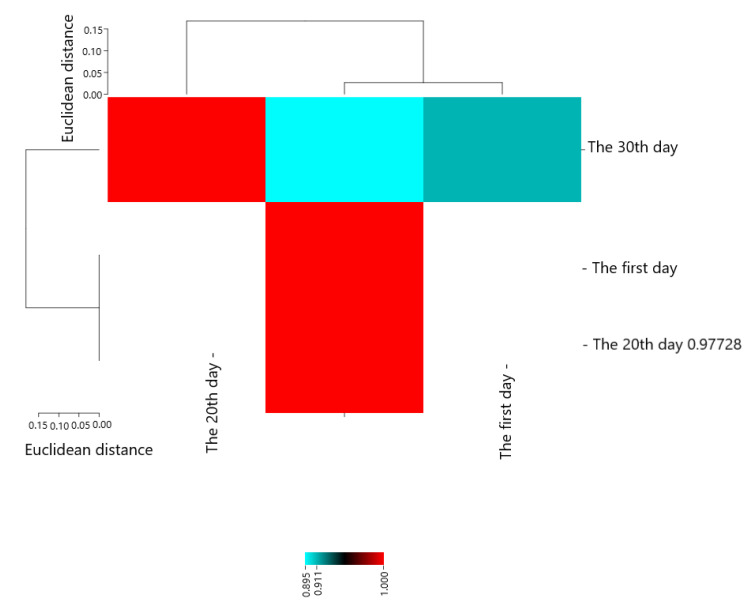
Heat map of mutual relations of chitosan and essential oil on *S. aureus* (cfu/g) in cheese during storage in correlation coefficient.

Pearson’s correlation coefficients are presented in Figure 3. The results showed that The first day with The 20th day (0.97726 **), The 30th day (0.89521 **), and The 20th day with The 30th day (0.91058 *) had the most positive and significant correlation.

### 2.6. The Effect of Various Concentrations of Chitosan and Pulegone on S. aureus (cfu/g) in Cheese during Storage

The results of Table 11 show that by increasing the concentration of chitosan and pulegone in coatings, the number of *S. aureus* bacteria in cheese decreased. At the conclusion of the storage time, the smallest and the largest of the bacterial count were observed in the treatment coated with 10% chitosan and 50 ppm pulegone and the control, respectively. Increasing the storage period led to a decrease in the bacterial population in all treatments.

**Figure 4:**
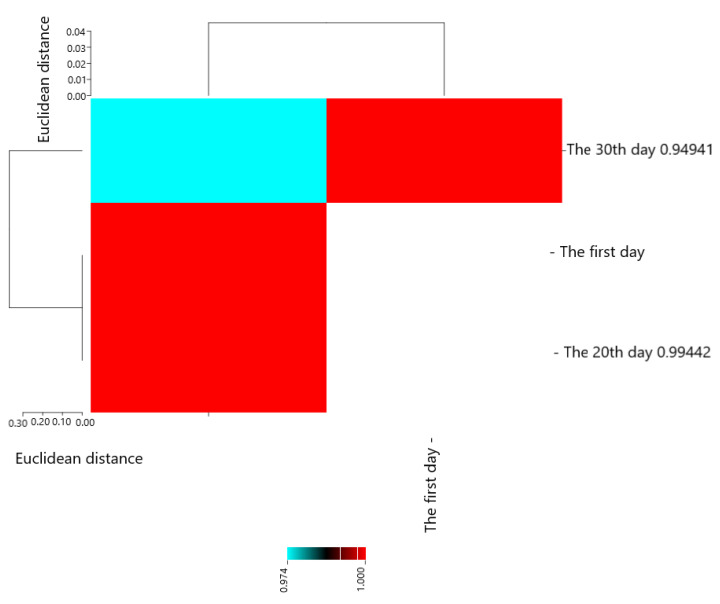
Heat map of mutual relations of chitosan and pulegone on *S. aureus* (cfu/g) in cheese during storage in correlation coefficient.

Pearson’s correlation coefficients are presented in Figure 4. The results showed that The first day with The 20th day (0.99442 **), The 30th day (0.94941 **), and The 20th day with The 30th day (0.97392 **) had the most positive and significant correlation.

### 2.7. The Effect of Various Concentrations of Chitosan and M. longifolia Essential Oil on L. monocytogenes (cfu/g) in Cheese during Storage

The results of Table 12 show that the number of *L. monocytogenes* in cheese decreased by increasing the storage period in all treatments. At the end of the storage time, the highest amount of bacterial count was observed in the control, which had no significant difference with the treatment coated with 10% chitosan without essential oil. By increasing the essential oil concentration in the coatings, the number of *L. monocytogenes* decreased. Increasing the concentration of chitosan also led to a decrease in the bacterial population.

**Figure 5:**
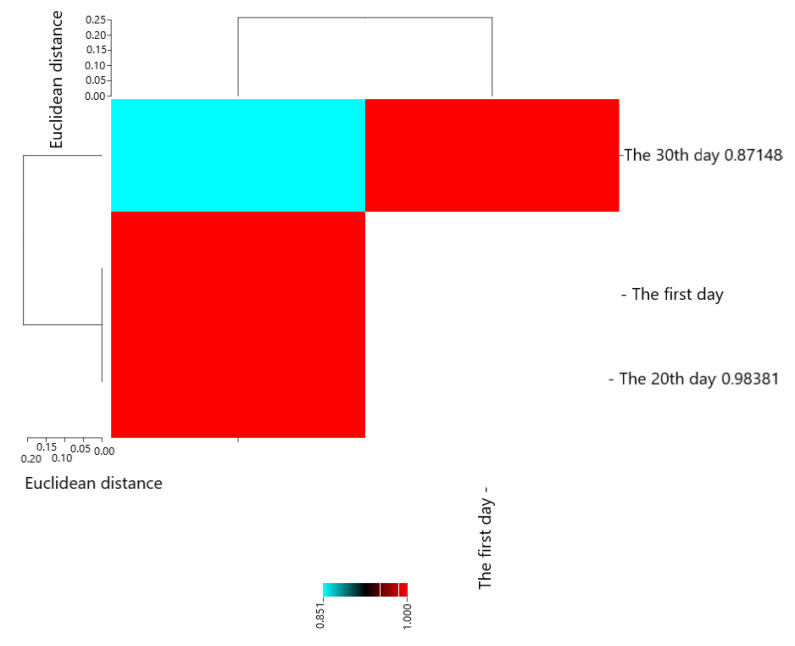
Heat map of mutual relations of chitosan and essential oil on *L. monocytogenes* (cfu/g) in cheese during storage in correlation coefficient.

Pearson’s correlation coefficients are presented in Figure 5. The results showed that The first day with The 20th day (0.98381 **), The 30th day (0.87148 **), and The 20th day with The 30th day (0.85096 *) had the most positive and significant correlation.

### 2.8. The Effect of Various Concentrations of Chitosan and Pulegone on L. monocytogenes (cfu/g) in Cheese during Storage

Table 13’s results show that at the conclusion of the storage period, the treatment coated with 10% chitosan and 50 ppm pulegone had the lowest number of bacteria, which had no significant difference with the treatments coated with 10% chitosan and 25 ppm pulegone and the treatment coated with 10% chitosan and 10 ppm pulegone (*p*> 0.05). Over the course of all storage times, the control treatment had the highest bacterial count. On the first and 20th day, at 5% and 10% chitosan concentrations, increasing the amount of pulegone in the coatings did not have a significant effect on the bacterial population. On the 30th day, in the concentration of 10% chitosan, increasing the pulegone concentration from 10 to 50 ppm did not have a significant effect on the number of *L. monocytogenes*.

**Figure 6:**
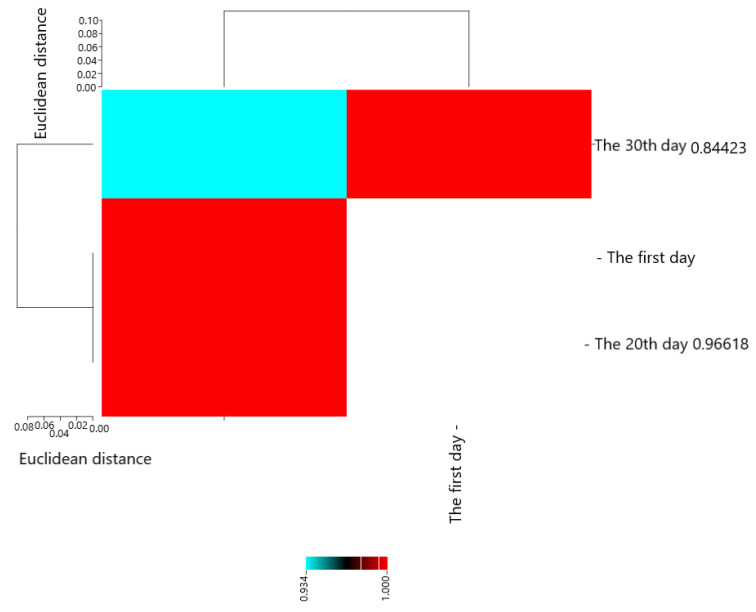
Heat map of mutual relations of chitosan and pulegone on *L. monocytogenes* (cfu/g) in cheese during storage in correlation coefficient.

Pearson’s correlation coefficients are presented in Figure 6. The results showed that The first day with The 20th day (0.96618 **), The 30th day (0.84423 *), and The 20th day with The 30th day (0.93431 **) had the most positive and significant correlation.

## 3. Material and Methods

Materials used in the study were *S. aureus* ATCC 29213, and *L. monocytogenes* ATCC 19,115 (from the fungus and bacteria collection center of the Iranian Scientific and Industrial Research Organization); *E. coli* O157 ATCC 43,895 (from the Department of Microbiology, Faculty of Veterinary Medicine, University of Tehran); Sodium alginate (Sigma-Aldrich, St. Louis, MO, USA); chitosan with a deacetylation level of greater than 75% (Sigma-Aldrich, St. Louis, MO, USA); Tryptic Soy Broth (TSB); Baird parker agar; Sorbitol-MacConkey agar; Palcam Listeria-Selective agar culture media (Merck, Darmstadt, Germany); and pulegone active ingredient (99.9% purity, Barij Essence Pharmaceutical Company, Kashan, Iran).

### 3.1. Collection of Plants and Production of Essential Oils

After the scientific confirmation of the species by plant science experts at University of Jiroft Herbarium, *M. longifolia* was taken in May 2021 from a cold area of Jiroft City. The aerial part of this plant was dried in the shade and ambient temperature. After drying, the plant was ground. About 100 g of dried sample was placed in 400 mL distilled water and submitted to hydrodistillation for 3 h using a Clevenger-type apparatus [24]. The yield of essential oil extraction from *M. longifolia* was 1%(*v*/*w*), and it was colorless. Gas chromatography (Shimadzu single quadrupole GCMS-QP2010 SE, Kyoto, Japan) equipped with a mass spectrometer (GC-MS) was used to determine the chemical composition of *M. longifolia* essential oil. Compounds were separated on HP-5 MS capillary column (30 m × 0.25 mm, film thickness 0.25 μm; Little Falls). A sample of 1.0 μL was injected in the split mode with split ratio 1:100. Helium was used as a carrier gas at a flow rate of 1.0 mL/min. The injection temperature was 230 °C. Compounds were further identified and authenticated using their complete mass fragmentation data compared to the NIST02.L and WILEY7n.L mass spectral libraries and published mass spectra and, wherever possible, by coinjection with authentic standards [24].

### 3.2. Preparation of Cheese

Lactic cheese samples were prepared at Pegah Dairy Company of Jiroft. The steps were as follows:

First, raw milk was standardized (the amount of fat (3%) and dry matter (15%) was adjusted). Then the milk temperature rose to 96 °C. A mixture of sour yogurt and vinegar was added to the hot stirring milk until the milk was completely coagulated. The clot was cut, drained, and poured into plastic molds, and within 5 h, the molds were returned to complete dehydration. Cheese pieces were placed in salt solution (16%) at 5 °C, and after 72 h, the relevant tests (pH, moisture content, salt content) were performed on them [16]. The pH, moisture, and salt content of the cheese samples were 5.5, 65, and 4%, respectively [25].

### 3.3. Treatment of Cheese Samples

The cheese samples were cut into cubes (length, width, and height of 3 cm) and coated by immersion method, during which the samples were immersed in the coating mixtures for 1 min until all surfaces of the cheese samples were completely covered with the coating material. Coating mixtures were sodium alginate solution (25%) with concentrations of 0, 5, and 10% chitosan and different concentrations of *M. longifolia* essential oil (0, 100, and 150 ppm) as well as different concentrations of pulegone (10, 25, and 50 ppm). The samples were then placed in an incubator (Fan Azma Gostar, Tehran, Iran) under controlled temperature and humidity (about 12 °C and relative humidity of 85%) for approximately 8 h until the coatings were dry [26].

In the pre-test, we used higher concentrations of *M. longifolia* essential oil and pulegone in the coatings, and a sensory evaluation test was also performed. The maximum concentration of *M. longifolia* essential oil and pulegone was determined according to the results obtained from the sensory evaluation. By increasing the concentration more than 150 ppm for *M. longifolia* essential oil and 50 ppm for pulegone, the flavor and taste scores decreased. In this way, the studied concentrations were determined for essential oil and pulegone.

### 3.4. Inoculation of the Desired Bacteria into Cheese Samples

To inoculate the bacteria (*S. aureus*, *L. monocytogenes*, and *E. coli O157*) into the cheese texture, bacterial suspensions containing 10^5^ CFU/g were injected into 8 points of cheese samples with a sterile syringe. After that, the samples were placed in polypropylene containers and kept at 5 °C [27]. Sampling and culture were performed once every 10 days for 1 month.

### 3.5. Bacterial Count in Cheese Samples

In order to count the bacteria, 1 g of the cheese sample was thoroughly homogenized in 9 mL of physiological saline. Then, 0.1 mL of this solution was cultured on specific media for each bacterium.

Baird Parker agar was used to count *S. aureus* at 37 °C for 24–48 h [28]. Sorbitol-MacConkey agar was used to count *E. coli* O157 bacteria at 37 °C for 24–48 h [29]. Palcam Listeria-Selective agar was used to count *L. monocytogenes* at 36 °C for 24–48 h [30].

### 3.6. Statistical Analysis

Analysis of variance (ANOVA) was used with the Statistix ver. 10 software to see whether there were any significant differences between the results. Differences at *p* ≤ 0.05 were considered significant. All experiments were performed in triplicate.

## 4. Discussion

Cheese is a ready-to-eat food product that is not subjected to any other treatment to ensure its safety before consumption. Contamination of cheese with foodborne pathogens may occur in several stages (before production, during production, and during storage period). Therefore, information on the main sources of pathogens and the mechanisms by which they infect the dairy chain is needed if contamination of any cheese is to be prevented [31]. The use of different additives in cheese can partially inhibit its bacterial population. Essential oils, extracts, and powders of herbs are compounds that can be added to cheese to reduce microbial contamination and increase sensory properties. In this study, the effect of an edible coating containing alginate, chitosan (0, 5, and 10%), *M. longifolia* essential oil (0, 100, and 150 ppm), and pulegone (0, 10, 25, and 50 ppm) on the growth of *E. coli*, *S. aureus*, and *L. monocytogenes* in cheese was examined during three storage times. In general, by increasing the storage period (30 days) in all treatments, the number of bacteria in the cheese decreased. This decrease was more pronounced in treatments with increasing concentrations of chitosan, essential oil, and pulegone. The effect of the studied treatments on reducing the growth of *E. coli* was greater than the other two bacteria. In general, the pulegone active ingredient was more effective in reducing the growth of bacteria than the *M. longifolia* essential oil.

Studies have demonstrated the antibacterial properties of chitosan, *M. longifolia* essential oil, and the pulegone active component. Evaluation of the antimicrobial effects of *M. longifolia* essential oil against *E. coli*, *S. aureus*, and *Candida albicans* showed that the pulegone and 1,8-cineole compounds are important in this regard [32]. In our previous study on the same essential oil, the compounds of piperitenone oxide (26.07%), pulegone (19.72%), piperitenone (11.88%), and 1,8-cineole (8.21%) were the major compounds present in the essential oil that can play a very important role in its antimicrobial activity [33]. *M. longifolia* essential oil has been found to have strong antibacterial properties against a variety of bacteria, including *Staphylococcus*, *Pseudomonas*, *Bacillus*, and *E. coli*, as well as some fungal strains including *Aspergillus*, *Fusarium*, and *Penicillium* [34].

It has been reported that one of the key characteristics of essential oils and active substances is the hydrophobic property, which led to the change and destruction of the cell membrane structure and their greater permeability. This is concerning the action of these substances and their compounds in the death of pathogenic bacteria. The result is that the majority of the ions and other essential components of the cell leak out, which ultimately causes the bacterium’s death [35]. It will lead to defects in the synthesis of many cell-wall polysaccharide compounds, and inhibit cell growth and morphogenesis [36]. The antimicrobial performance of essential oils in vitro depends on various factors such as antimicrobial components, type of microorganism, culture medium, amount of inoculum, pH, temperature, and food composition [37,38]. It was related [39] that, in a study on the antibacterial effect of *M. longifolia* essential oil against several foodborne pathogens, pulegone, 1,8-cineole, and menthofuran were the most prevalent constituents of essential oils. Their results also showed that the most sensitive bacterium to *M. longifolia* essential oil was *E. coli*, which is consistent with our results. In another study, it was found that *M. longifolia* from the mint family had antibacterial properties on *Staphylococcus* and *Listeria* species. The results of this study similarly confirm the results of our study [40]. Studies have shown that the effect of different essential oils is concentration-dependent so that in low concentrations, phenolic compounds act on the enzymatic activity, particularly those involved in energy generation, but in high concentrations these can cause protein denaturation [41]. In one study, the antibacterial effects of pulegone and 1,8-cineole against *S. aureus* and *Salmonella typhimurium* were investigated, and the results showed that 1,8-cineole has a stronger antibacterial effect on Gram-positive bacteria than Gram-negative bacteria, while pulegone has a higher antibacterial effect on Gram-negative bacteria [42], which is consistent with the results of the present study.

Comparing the results of *M. longifolia* essential oil and pulegone as the effective ingredient and the main composition of *M. longifolia* essential oil, it was found that this substance had more antibacterial properties than the complete essential oil in much lower concentrations. Pulegone is a monoterpene ketone found in the leaves and flowers of several members of the mint family [16]. Terpenes are capable of penetrating the bacterial cell wall, leading to the denaturation of proteins and disintegration of the cell membrane, leading to cytoplasmic leakage, cell lysis, and eventually cell death [43]. Based on the published reports, pulegone can effectively destroy *S. aureus, S. typhimurium*, and *E. coli* [44].

The antibacterial effects of trans-cinnamaldehyde, 1,8-cineole, and pulegone against *Streptococcus equi* subsp. equi were investigated [45]. According to the results, trans-cinnamaldehyde, 1,8-cineole, and pulegone possess antibacterial capabilities and may serve as a convenient and reasonably priced alternative to synthetic antibiotics. According to the results of the current study, chitosan in edible coatings inhibited bacterial growth as compared to the control, and antibacterial activity improved by increasing chitosan content.

In general, the antimicrobial activity of essential oils is expressed by several mechanisms:

1. Dissolving in the cytoplasmic membrane and interfering with the protein structure of the enzyme and destroying the microorganism;
2. Interruption in activities related to succinate and reactions related to NADH;
3. Disturbing electron transfer in the respiratory chain;
4. Creating a break in oxidative phosphorylation.

The lipophilic property of essential oils can explain its increased membrane permeability or destruction due to the activity of enzymes in the cell membrane such as protein kinase [46].

Chitosan’s polycationic composition is thought to be the source of its antibacterial properties. The protonated amino group in chitosan interacts electrostatically with the negative residues on cellular surfaces to achieve antibacterial activity [47]. With increasing levels of deacetylation, chitosan contains more protonated amino groups, which affects its antibacterial efficacy [48]. The growth of aerobic bacteria can be inhibited by coverings made of chitosan. Chitosan coatings keep oxygen away from pathogenic bacteria because they block air penetration [49,50]. The antibacterial properties of chitosan film enhanced with oregano and thyme essential oils have been investigated [51]. Their results showed that even at the lowest concentrations, the chitosan film with essential oils may suppress bacterial and fungal development. In comparison to the doses required to stop the development of the beneficial bacteria of *Lactobacillus rahmnosu* and *Enterococcus faecium*, all antimicrobial agents’ MIC and MBC against *E. coli* and *S. aureus* were extremely low.

In some studies [52,53,54], the antibacterial effectiveness of chitosan as an edible coating against *L. monocytogenes* on the surface of ready-to-eat roast beef was examined. Results showed that *L. monocytogenes* on the surface of roast beef may be controlled by chitosan coatings. In [55], the effects of an edible chitosan coating on the quality and shelf life of sliced mango fruit were investigated. Mango slices were exposed to aqueous solutions containing 0%, 0.5%, 1%, and 2% chitosan. The results showed that coating with chitosan efficiently stopped the growth of microorganisms.

## 5. Conclusions

In this research, the impact of edible coatings of alginate and chitosan along with *M. longifolia* essential oil and the active ingredient of pulegone on the growth of some pathogenic bacteria in lactic cheese was investigated. The results generally demonstrated that the population of investigated microorganisms decreased by increasing the concentration of chitosan, *M. longifolia*, and pulegone in edible coatings. When the effects of pulegone and *M. longifolia* essential oils on bacteria were compared, it was found that pulegone had a stronger impact on bacterial population reduction. Coating treatments showed more antibacterial activity on *E. coli* than other bacteria. From this study, it can be concluded that chitosan coating along with *M. longifolia* essential oil and its active ingredient pulegone had antibacterial effects against *S. aureus*, *L. monocytogenes*, and *E. coli* in cheese. As a result, it can be utilized as a strong and natural food preservative.

## Figures and Tables

**Table 1:** Chemical composition of *M. longifolia* essential oil.

No.	Compounds	Retention Index	Amount (%)	Chemical Type
1	α-Pinene	935	0.4	monoterpenes
2	Sabinene	971	0.52	monoterpenes
3	Camphene	975	0.11	monoterpenes
4	β-Pinene	979	0.78	monoterpenes
5	3-Octanol	991	0.67	etc.
6	Myrcene	997	0.49	monoterpenes
7	Nitrophthalic acid	1017	2.14	etc.
8	Limonene	1026	1.42	monoterpenes
9	Ocimene	1034	0.16	monoterpenes
10	1,8-Cineole	1037	8.21	monoterpenes
11	Isoamyl 2-methylbutyrate	1087	0.17	etc.
12	Linalol	1101	0.14	monoterpenes
13	Menthone	1156	1.85	monoterpenes
14	Terpenol	1179	0.57	monoterpenes
15	4-Terpineol	1197	0.80	monoterpenes
16	Pulegone	1250	26.07	monoterpenes
17	Thymol	1297	0.18	monoterpenes
18	L-Pinocarveol	1325	0.22	monoterpenes
19	β-Bisabolen	1336	0.29	sesquiterpenes
20	piperitenone oxide	1345	19.72	monoterpenes
21	piperitenone	1359	11.88	monoterpenes
22	cis-piperitone oxide	1365	6.35	etc.
23	Buchu camphor	1367	0.25	diosphenol
24	Piperitone	1369	3.22	monoterpenes
25	Isophorone	1384	0.94	phenol
26	Dimethylcyclohexanone	1402	0.28	etc.
27	Caryophyllene	1414	0.80	sesquiterpenes
28	Germacrene D	1458	0.29	monoterpenes
29	3-Nonanol	1509	0.22	etc.
30	Camphor	1532	0.22	monoterpenes
31	cis-isopulegone	1576	0.82	monoterpenes
32	Spathulenol	1578	0.17	sesquiterpenes
33	Caryophyllene oxide	1582	0.19	sesquiterpenes
34	Pinocarvone	1586	0.10	etc.
35	DHPM	1596	0.12	etc.
36	2-Methylpyrimidine-4,6-diol	1599	0.2	etc.
37	Benzylacetone	1621	0.25	etc.
38	Borneol	1719	5.96	monoterpenes
Total	-		97.8	

**Table 2:** Descriptive statistics related to the chitosan and essential oil on *E. coli* (cfu/g) in cheese during storage.

Trait	Mean	Range	Standard Variation	Minimum	Maximum
The first day	9101	7354	2045	6846	14,200
The 20th day	4867	3503	1065	3700	7203
The 30th day	3685	2966	830	2800	5766

**Table 3:** Descriptive statistics related to the chitosan and pulegone on *E. coli* (cfu/g) in cheese during storage.

Trait	Mean	Range	Standard Variation	Minimum	Maximum
The first day	3603	6102	1339	883.33000	14,200
The 20th day	2111	6570	1018	563.33000	7133
The 30th day	1530	5430	886	303.33000	5733

**Table 4:** Descriptive statistics related to the chitosan and essential oil on *S. aureus* (cfu/g) in cheese during storage.

Trait	Mean	Range	Standard Variation	Minimum	Maximum
The first day	219,720	211,200	65,016	110,466	321,666
The 20th day	198,307	181,334	58,834	84,666	266,000
The 30th day	133,760	114,333	34,529	60,333	174,666

**Table 5:** Descriptive statistics related to the chitosan and pulegone on *S. aureus* (cfu/g) in cheese during storage.

Trait	Mean	Range	Standard Variation	Minimum	Maximum
The first day	98,783	287,666	67,427	34,000	321,666
The 20th day	83,096	247,000	57,503	19,000	266,000
The 30th day	72,911	159,266	38,038	15,400	174,666

**Table 6:** Descriptive statistics related to the chitosan and essential oil on *L. monocytogenes* (cfu/g) in cheese during storage.

Trait	Mean	Range	Standard Variation	Minimum	Maximum
The first day	47,698	115,240	32,707	11,093	126,333
The 20th day	26,593	46,566	14,483	9100	55,666
The 30th day	12,006	15,274	4526	5726	21,000

**Table 7:** Descriptive statistics related to the chitosan and pulegone on *L. monocytogenes* (cfu/g) in cheese during storage.

Trait	Mean	Range	Standard Variation	Minimum	Maximum
The first day	20,534	124,434	11,338	1899	126,333
The 20th day	12,832	54,209	10,863	1457	55,666
The 30th day	7854	19,890	6267	1110	21,000

**Table 8:** Effect of chitosan and essential oil on *E. coli* (cfu/g) in cheese during storage.

Treatments	The First Day	The 20th Day	The 30th Day
Chitosan (0) essential oil (0): Control	14,200.00 ^a,^*	7133.33 ^a^	5733.33 ^a^
Chitosan (0) essential oil (100 ppm)	9300.00 ^bcd^	5750.00 ^b^	3373.33 ^cd^
Chitosan (0) essential oil (150 ppm)	8916.66 ^bcd^	5680.00 ^b^	3416.66 ^cd^
Chitosan (5%) essential oil (0)	11,033.33 ^b^	4966.67 ^bc^	4533.33 ^b^
Chitosan (5%) essential oil (100 ppm)	8144.33 ^cd^	4286.67 ^c^	3253.33 ^d^
Chitosan (5%) essential oil (150 ppm)	7383.33 ^d^	4313.33 ^c^	3113.33 ^d^
Chitosan (10%) essential oil (0)	10,425.00 ^bc^	4596.67 ^bc^	4386.66 ^bc^
Chitosan (10%) essential oil (100 ppm)	7366.66 ^d^	4143.33 ^c^	3166.66 ^d^
Chitosan (10%) essential oil (150 ppm)	6846.66 ^d^	3716.67 ^c^	2883.33 ^d^

* In each column, the numbers with the same letters had no significant difference (*p* > 0.05).

**Table 9:** Effect of chitosan and pulegone on *E. coli* (cfu/g) in cheese during storage.

Treatments	The First Day	The 20th Day	The 30th Day
Chitosan (0) pulegone (0): Control	14,200.00 ^a,^*	7133.33 ^a^	5733.33 ^a^
Chitosan (0) pulegone (10 ppm)	8266.67 ^b^	4633.33 ^b^	2850.00 ^b^
Chitosan (0) pulegone (25 ppm)	3500.00 ^c^	2673.33 ^c^	1960.00 ^c^
Chitosan (0) pulegone (50 ppm)	3100.00 ^cd^	1833.33 ^cd^	726.67 ^de^
Chitosan (5%) pulegone (10 ppm)	1866.67 ^cd^	1840.00 ^cd^	1766.67 ^c^
Chitosan (5%) pulegone (25 ppm)	1460.00 ^cd^	1329.67 ^d^	1081.67 ^d^
Chitosan (5%) pulegone (50 ppm)	1100.00 ^d^	1083.33 ^d^	913.33 ^de^
Chitosan (10%) pulegone (10 ppm)	966.67 ^d^	910.00 ^d^	770.00 ^de^
Chitosan (10%) pulegone (25 ppm)	946.67 ^d^	796.67 ^d^	603.33 ^de^
Chitosan (10%) pulegone (50 ppm)	883.33 ^d^	563.33 ^d^	303.33 ^e^

* In each column, the numbers with the same letters had no significant difference (*p* > 0.05).

**Table 10:** Effect of chitosan and essential oil on *S. aureus* (cfu/g) in cheese during storage.

Treatments	The First Day	The 20th Day	The 30th Day
Chitosan (0) essential oil (0): Control	321,666.67 ^a,^*	266,000.00 ^a^	174,666.67 ^a^
Chitosan (0) essential oil (100 ppm)	264,000.00 ^c^	254,666.67 ^a^	135,666.67 ^b^
Chitosan (0) essential oil (150 ppm)	251,666.67 ^c^	219,666.67 ^b^	132,666.67 ^b^
Chitosan (5%) essential oil (0)	291,666.67 ^b^	260,666.67 ^a^	172,333.33 ^a^
Chitosan (5%) essential oil (100 ppm)	192,666.67 ^d^	175,000.00 ^c^	134,666.67 ^b^
Chitosan (5%) essential oil (150 ppm)	167,000.00 ^e^	166,000.00 ^cd^	121,150.00 ^b^
Chitosan (10%) essential oil (0)	252,666.67 ^c^	233,000.00 ^b^	171,333.33 ^a^
Chitosan (10%) essential oil (100 ppm)	159,666.67 ^e^	147,666.67 ^d^	114,666.67 ^b^
Chitosan (10%) essential oil (150 ppm)	110,466.67 ^f^	84,666.67 ^e^	60,333.33 ^c^

* In each column, the numbers with the same letters had no significant difference (*p* > 0.05).

**Table 11:** Effect of chitosan and pulegone on *S. aureus* (cfu/g) in cheese during storage.

Treatments	The First Day	The 20th Day	The 30th Day
Chitosan (0) pulegone (0): Control	321,666.66 ^a,^*	266,000.00 ^a^	174,666.67 ^a^
Chitosan (0) pulegone (10 ppm)	133,000.00 ^b^	113,333.33 ^b^	103,366.67 ^b^
Chitosan (0) pulegone (25 ppm)	106,133.33 ^c^	100,333.33 ^c^	95,333.33 ^b^
Chitosan (0) pulegone (50 ppm)	79,666.67 ^d^	71,100.00 ^ef^	69,970.00 ^cd^
Chitosan (5%) pulegone (10 ppm)	106,333.33 ^c^	87,633.33 ^d^	80,800.00 ^c^
Chitosan (5%) pulegone (25 ppm)	82,666.67 ^d^	78,033.33 ^de^	74,333.33 ^c^
Chitosan (5%) pulegone (50 ppm)	68,666.67 ^de^	62,166.67 ^fg^	59,033.33 ^de^
Chitosan (10%) pulegone (10 ppm)	68,333.33 ^de^	51,000.00 ^gh^	49,966.67 ^ef^
Chitosan (10%) pulegone (25 ppm)	61,666.67 ^e^	43,333.33 ^h^	40,166.67 ^f^
Chitosan (10%) pulegone (50 ppm)	34,000.00 ^f^	19,000.00 ^i^	15,400.00 ^g^

* In each column, the numbers with the same letters had no significant difference (*p* > 0.05).

**Table 12:** Effect of chitosan and essential oil on *L. monocytogenes* (cfu/g) in cheese during storage.

Treatments	The First Day	The 20th Day	The 30th Day
Chitosan (0) essential oil (0): Control	126,333.33 ^a,^*	55,666.67 ^a^	21,000.00 ^a^
Chitosan (0) essential oil (100 ppm)	63,333.33 ^c^	35,000.00 ^b^	13,000.00 ^bc^
Chitosan (0) essential oil (150 ppm)	36,666.67 ^d^	19,700.00 ^cd^	8666.67 ^de^
Chitosan (5%) essential oil (0)	84,333.33 ^b^	47,666.67 ^a^	14,000.00 ^b^
Chitosan (5%) essential oil (100 ppm)	28,066.67 ^de^	19,333.33 ^cd^	10,333.33 ^cd^
Chitosan (5%) essential oil (150 ppm)	20,000.00 ^ef^	15,366.67 ^d^	10,200.00 ^cd^
Chitosan (10%) essential oil (0)	59,333.33 ^c^	31,333.33 ^bc^	18,900.00 ^a^
Chitosan (10%) essential oil (100 ppm)	26,340.00 ^de^	15,866.67 ^d^	9233.33 ^d^
Chitosan (10%) essential oil (150 ppm)	11,093.33 ^f^	9100.00 ^d^	5726.67 ^e^

* In each column, the numbers with the same letters had no significant difference (*p* > 0.05).

**Table 13:** Effect of chitosan and pulegone on *L. monocytogenes* (cfu/g) in cheese during storage.

Treatments	The First Day	The 20th Day	The 30th Day
Chitosan (0) pulegone (0): Control	126,333.33 ^a,^*	55,666.67 ^a^	21,000.00 ^a^
Chitosan (0) pulegone (10 ppm)	35,833.33 ^b^	30,333.33 ^b^	15,333.33 ^b^
Chitosan (0) pulegone (25 ppm)	28,000.00 ^b^	19,333.33 ^c^	15,000.00 ^b^
Chitosan (0) pulegone (50 ppm)	13,666.67 ^c^	11,133.33 ^d^	8966.67 ^c^
Chitosan (5%) pulegone (10 ppm)	13,666.67 ^c^	9266.67 ^d^	8366.67 ^c^
Chitosan (5%) pulegone (25 ppm)	9100.00 ^cd^	6900.00 ^d^	5566.67 ^d^
Chitosan (5%) pulegone (50 ppm)	8133.33 ^cd^	5470.00 ^de^	4700.00 ^d^
Chitosan (10%) pulegone (10 ppm)	1998.67 ^d^	1577.00 ^e^	1110.00 ^e^
Chitosan (10%) pulegone (25 ppm)	1979.67 ^d^	1466.67 ^e^	1537.33 ^e^
Chitosan (10%) pulegone (50 ppm)	1899.33 ^d^	1457.67 ^e^	1347.33 ^e^

* In each column, the numbers with the same letters had no significant difference (*p* > 0.05).

## Data Availability

All the data are embedded in the manuscript.
